# New York Odontological Society

**Published:** 1886-07

**Authors:** 


### NEW YORK ODONTOLOGICAL SOCIETY.

REPORTED EXPRESSLY FOR THE INDEPENDENT PRACTITIONER.

This society held its last meeting of the season in the parlors of the Academy of Medicine, Tuesday evening, June 8th, President Bogue in the chair.

Dr. N. W. Kingsley, with a drawing on the blackboard, described a prosthetic appliance devised and made by Dr. A. Rosenthal, of Liege, Belgium. The patient for whom the appliance was constructed had lost a number of teeth on opposite sides of each jaw, in consequence of which the lower jaw had become turned or drawn by the muscles greatly to one side, thus interfering with, or making mastication difficult, and presenting an exceedingly ugly appearance. To correct this, a band was made to fit around a number of the upper teeth and held firmly in position by screws. To the band was attached a rod directed downward. A similar band was prepared and attached to the lower teeth, with a small tube pointing upward. The jaw was brought into position, and with the appliance adjusted the rod from the upper band played up and down in the tube of the lower as the mouth opened and shut, still retaining the position. In this way mastication could be fairly well performed, though it was impossible to obtain lateral movement.

Dr. Davenport, the Secretary, read a paper prepared and sent by Dr. Wm. Herbert Rollins, of Boston, entitled "Regular Examination of the Saliva." In this the doctor called attention to the benefits secured by watching the condition of the saliva, which often changed with the varying conditions of the system. When strongly alkaline in character, beneficial results might be obtained by acid treatment, or when found of a decidedly acid nature the reverse treatment was called for.

For examining and testing the saliva, prepared litmus paper was now offered at the dental depots, thus enabling every dentist to readily ascertain the condition of the saliva in each case presented.

Dr. Geo. W. Weld, read an exceedingly interesting paper on "The Destructive Energy of Tincture Chloride of Iron on the Teeth." The doctor had performed a series of experiments for the purpose of ascertaining the effect of various acids upon lime, and presented to the society a handsomely framed card containing specimens of teeth which had been subjected to iron and acid preparations, such as physicians commonly prescribe. Dr. Weld also passed around the room, for the inspection of the members present, trade bottles of acid phosphates of iron in common use. He had submerged teeth in these different preparations, and in several of them the enamel was much eroded, and the entire dentinal structure presented the appearance of having been baked. Teeth subjected to the phospho-muriate of quinine preparations, though containing phosphoric acid, iron and strychnine with the quinine, were not affected by them, and this Dr. Weld attributed to the fact that the preparation was in the form of *syrup*, and so he considered it safer to administer iron in syrup.

It was asked why the syrup should prevent deleterious action, but the doctor could only suggest that the teeth possibly were better covered or protected by the syrup, which prevented the acid or iron from acting on them. Dr. Weld thought much injury was done to the teeth by physicians, who prescribed improperly prepared iron and acid medicines.

An elaborate chart was exhibited showing the different preparations of iron and their compounds; also preparations of phosphates, phosphites, and hypo-phosphites.

A vote of thanks was tendered by the society to Dr. Weld, for the great care he had given to, and the time expended on this important subject.

